# Effectiveness of Foot Biomechanical Orthoses to Relieve Patients Suffering from Plantar* Fasciitis*: Is the Reduction of Pain Related to Change in Neural Strategy?

**DOI:** 10.1155/2018/3594150

**Published:** 2018-12-12

**Authors:** Sébastien Moyne-Bressand, Carole Dhieux, Erick Dousset, Patrick Decherchi

**Affiliations:** Aix-Marseille Université, CNRS, UMR 7287 Institut des Sciences du Mouvement: Etienne-Jules MAREY (ISM), Equipe Plasticité des Systèmes Nerveux et Musculaire (PSNM), Parc Scientifique et Technologique de Luminy, Faculté des Sciences du Sport de Marseille, CC910 - 163 avenue de Luminy, 13288 Marseille Cedex 09, France

## Abstract

Plantar* fasciitis* is a cause of chronic pain under the heel and bottom of the foot. One of the treatments to reduce pain consists of using plantar orthoses to address specific imbalances during foot placement or gait. The aim of the present study was to determine if reduction of pain with a treatment based on plantar orthoses is related to changes in reflexes and muscle activity of the muscles of the lower limbs. Ten patients (51.0±3.5 years, 76.0±2.38 kg, 171.9±1.8 cm, 3 women and 7 men) with plantar* fasciitis* for less than 1 year were followed up during nine weeks.* Soleus* M, H, and V waves recorded at rest and during voluntary contraction and Root Mean Square-Electromyogram from four leg muscles recorded during walking and static position were analyzed in patients before and 3, 6, and 9 weeks after wearing orthoses. Pain level and gait and posture parameters were also analyzed. Results were compared to five healthy participants exhibiting no pain (30.6±2.1 years, 60.0±3.5 kg, 167.0±3.4 cm, 3 women and 2 men). Results indicated that pain was significantly reduced after 3 weeks. H_max_/M_max_ and H_sup_/M_sup_ ratios were significantly higher and M_Hmax_/M_max_ and M_Hsup_/M_sup_ were significantly lower in healthy participants compared to patients with plantar* fasciitis*. No difference in the V/M_sup_ ratio was found between groups. Furthermore, all other measured locomotor, stabilometric, and electromyographic parameters remained unchanged throughout the entire protocol. The reduction of pain is not related to change in neural activity suggesting that, after 9 weeks of wearing plantar orthoses, patients are not yet cured and return to physical activity should be delayed.

## 1. Introduction

Plantar* fasciitis* (also called neuritis, calcaneodynia, stone bruise, subcalcaneal bursitis or pain, calcaneal periostitis or enthesopathy, plantar fascia insertitis, jogger's heel, and heel spur syndrome) is one of the most common orthopaedic complaints resulting from plantar fascia (a fibrous membrane also called aponeurosis) damage or tear that may lead to chronic pain and stiffness in the bottom of the heel or mid-foot area [1, 2]. Plantar* fasciitis* may or may not be associated with inflammatory reaction [3, 4]. During locomotion and standing, plantar fascia acts as shock absorbers and supports the arch of the foot. It is an important static stabilizer of the longitudinal arch of the foot. However, when the pressure on the plantar ligament is important in overweight or obese people or increases with sudden weight gain (pregnant women, etc.) or with repetitive activity (long-distance runner, activity involving being on feet often, etc.) the risk factor related to this overuse increases [5–7].

The initial treatments are to reduce activity and standing and possible inflammation by use of anti-inflammatory drugs in addition to using arch supports in the shoes. If pain continues and if all the conservative methods (corticosteroid injection, shock wave, ice therapy, electrostimulation, endoscopic plantar release, etc.) do not work or when symptoms worsen surgery may be considered [8, 9]. Surgery is effective in around 1% of patients who do not respond to conservative measures. Fortunately, it was reported that most patients respond to conservative treatment [10, 11]. Among the conservative management of plantar* fasciitis*, foot orthoses, that reduce tissue stress during standing and ambulation, and the ability to raise the medial longitudinal arch of the foot immediately relieve patients of pain [12, 13]. A lot of authors consider that plantar* fasciitis* results in an excessive foot pronation and suggest that subtalar joint pronation everts the calcaneus and lengthens the plantar aponeurosis, thereby increasing the intrafascial tension [12]. Cadaveric studies also reported that orthotic devices reduce the collapse of the longitudinal arch during static loading and elongation of the foot associated with pronation [12]. Thus, it was shown that foot orthosis is the best choice for initial treatment of plantar* fasciitis*. Indeed, pain is reduced, gait is improved, and walking distance is increased in individuals with plantar* fasciitis* wearing flat insoles [14–20].

Despite a variety of orthosis types available, it was shown that prefabricated heel pad was better or identical to customized orthoses for reducing symptom of plantar* fasciitis* than custom-made orthotic insert and that benefit can last up to 12 weeks [19]. Theoretically, orthotic device reduces pain by reducing and absorbing shocks that are normally absorbed by the plantar aponeurosis. In addition to changing cushioning, the role of foot orthoses is to change the distribution of charges and to reduce the torsion between the rear and forefoot and to stimulate the plantar sensor in order to correct the locomotion, postural deviations, and muscles deficiencies and to restore balance. Thus, foot orthoses work in two mechanisms: mechanical and proprioceptive corrections. In the first one, the in-shoe orthoses placed under the feet are designed to reduce abnormal foot movement according to the Root's Subtalar Joint Neutral Theory [21, 22], the Subtalar Joint Axis Location and Rotational Equilibrium Theory [23], the Physical Stress Theory [24], the Maximal Arch Subtalar Stabilization [25], or the Sagittal Plane Facilitation Theory [26]. Although a proprioceptive correction is also present, the mechanical one remains dominant [27]. The second strategy is mainly based on proprioceptive stimulations of the plantar sensors to modify the postural control [28]. In this case, the orthoses are placed under the feet according to the projection of the gravity center in order to change the tonus of muscle groups [27]. Although a mechanical correction is also present, the proprioceptive one remains dominant [27].

Because most studies were limited to evaluate Foot Function Index with a questionnaire and pain with a Visual Analogue Scale (VAS), the aim of the present study was to determine if reduction of pain with a treatment based on plantar orthoses known to correct postural deviations and muscle deficiencies and to address specific imbalances during foot placement or gait is related to changes in reflexes and muscle activity of the muscles of the lower limbs. Thus, neural strategy was accessed by firstly analyzing M(muscle)-, H(reflex)-, and V(volitional)-waves from* soleus* (SOL) muscle at rest and during voluntary contraction and Root Mean Square (RMS)-EMG from* vastus medialis* (VM),* tibialis anterior* (TA),* soleus*, and* biceps femoris* (BF) muscles in patients before and 3, 6, and 9 weeks after wearing orthoses. We hypothesized that plantar orthoses lead to spinal adaptations at the origin of the suppression of the pain. Furthermore, because we also hypothesized that potential changes in neural strategy could affect muscle activities of the lower limbs, gait (3 main steps of the stance phase) and posture (stance surface, Xm, Ym, LFS, and VFY parameters) were analyzed as a secondary measure. Results were compared to healthy participants exhibiting no pain. Analysis was performed over a period of nine weeks in order to characterize the middle term effects of the foot orthoses.

## 2. Materials and Methods

### 2.1. Subjects

This study was conducted in a podiatry office. Patients were included in the protocol from September 2014 to April 2016. For inclusion in this study, people had to suffer from chronic plantar pain for at least 6 months. Furthermore, no patients had had previous treatment of the injury. Only patients with a pain perception equal to or above 60% when active were included in the protocol. They had to be physically active, i.e., they had not been sedentary and they had regularly engaged, once a week, between 2 and 4 hours, in moderate levels of sport or physical or recreational activities. The diagnostic was an ultrasonography-verified chronic unilateral plantar* fasciitis* with* valgus* foot profile. They had to present pain at rest leading to impairment during their daily activities and increasing when the level of activity increases. No claudication was to be observed.

Exclusion criteria were infiltration and pharmacological treatments with anti-inflammatory drugs or corticosteroids, surgery, or any other treatments (ultrasound, electroshock, botulinum toxin injections, protein rich plasma injections, etc.). Furthermore, they were excluded if they had history of a major orthopedic or medical condition (inflammatory arthritis, diabetes, musculoskeletal complaints, sciatica, local nerve entrapment, spasticity, lumbar radiculitis, radiculopathy or myelopathy, history of foot or ankle fracture, systemic rheumatic disease, any lower extremity injury other than plantar* fasciitis*, etc.) that may have influenced the condition. Undiagnosed plantar* fasciitis* or nonambulatory and noncommunicative patients were also excluded. Finally, obesity patients with a Body Mass Index (BMI) greater than 30 kg.m^−2^ were excluded.

Eighteen patients were eligible. Four of them did not meet inclusion criteria and four declined to participate to the 9 weeks of follow-up. The remaining 10 patients (3 women and 7 men) suffering from plantar* fasciitis* participated in the entire study. They were compared to healthy participants (3 women and 2 men). For ethical considerations and because the French law does not allow, no eligible patient with plantar pain was enrolled in the study without treatment to constitute a SHAM group. Furthermore, sham devices like sham insoles previously used [15] could not be prescribed because such orthotics are not totally inert on the planter sensors and may have effects on limb muscle activities. Thus, following the initial appointment, subjects were allocated to only 2 groups (the healthy participants group called Control and the group of patients suffering from plantar pain and treated with orthotics).

**Figure 1:**
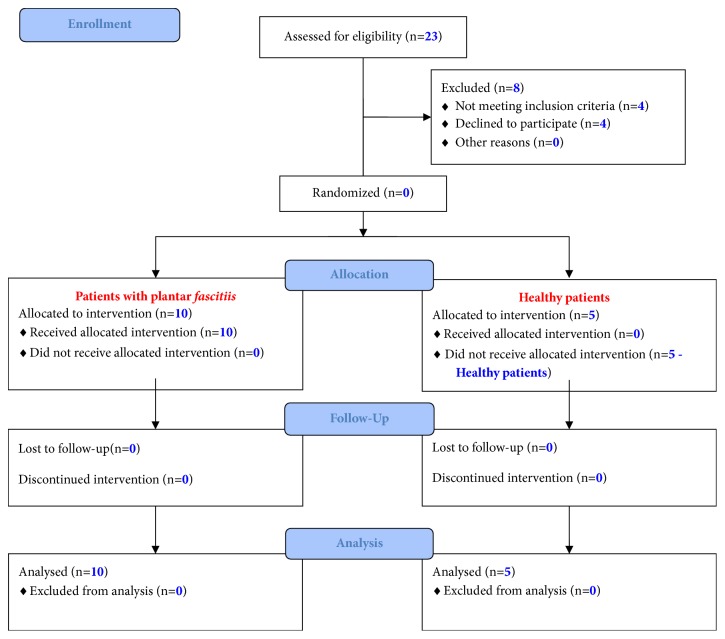
Flow diagram. The diagram shows the flow of the 2 groups of subjects through each stage of the trial.

Table 1 and Figure 1 show patient characteristics and the* flowchart of the 2 groups of subjects through each stage of the* trial, respectively.

### 2.2. Orthotics Prescription

Each subject was assessed using a standardized assessment by an experienced podiatrist. During the nine weeks of the study, custom-made plantar biomechanical podiatric orthoses, according to the instructions of the podiatrist, were bilaterally placed inside the shoes of patients suffering from plantar pain. During initial assessment, patients were positioned barefoot with a distance of 7 cm between feet. According to the Jean E. Smekens's vade-mecum, the foot was carefully measured by plantagraphy using a computer assisted optical podoscope and by making a mold of the foot using platform equipped with two silicone air bags. The aim was to respond to the specific needs of patients with pain, i.e., to reduce pain. The in-shoe orthoses are designed to reduce rearfoot misalignment and to hold the foot close to its subtalar neutral position (i.e., to resist to the excessive pronation of the foot) in the goal to restore normal alignment of the entire lower limb. Orthoses were fabricated in ethylene-vinyl acetate (EVA), an elastomeric copolymer with characteristic properties such as low-temperature toughness, stress-crack resistance, hot-melt adhesive waterproof properties, and resistance to UV radiation. The imprint of the sole was in EVA 50 Shore hardness while the corrective elements were in EVA 80 Shore hardness. Resin (1.2 mm thickness) was used to reinforce the heel and the inner arch of the sole. Finally, an absorbing material (3.2 mm thickness) was stuck under the sole at the level of the heel to absorb shock during locomotion. This relatively rigid device was designed to provide significant support for the foot and influence the position of the foot relative to the leg. The patients wore the custom-fit orthotics for activities of daily living at home and were instructed not to drastically alter the time they wore their shoes from a normal day. This device represents those that are commonly prescribed. Healthy participants did not wear foot orthoses.

### 2.3. Ethics, Consent, and Permissions

Eligible patients were informed about the study and the study design. They were also well informed about the advantages and disadvantages of participation. The experiments were conducted in accordance with the Declaration of Helsinki and were approved by the Regional Council of the College of podiatrists (trial registration: CRODP-PACA-A2013-04). All the subjects provided their written informed consent to participate in this study. Local ethics committee approved this consent procedure (IRB00005048).

### 2.4. Experimental Synopsis

The day of the first appointment, an examination was performed for the specific design of orthoses. Then, the experiment was divided into 4 sessions: at W0, two weeks later after the first appointment, orthoses were delivered and preorthotic scores were recorded and after three (W3), six (W6), and nine (W9) weeks wearing plantar orthoses. At each session, pain intensity, gait analysis coupled to EMG recordings, postural oscillations coupled to EMG recordings, and spinal reflexes and supraspinal influences at rest and during voluntary contraction of the* soleus* (SOL) muscle of the painful leg were recorded.

### 2.5. Experimental Protocol

*Pain*. As previously described [29], to analyze and quantify the effect of wearing orthoses, the pain experienced was measured at each experimental session using a Visual Analog Scale (VAS). The VAS is valid and reliable [30] and has already been used in several planar* fasciitis* studies [31].

*Locomotion*. Gait analysis was carried out on dynamic platform (RSscan® International, Footscan®, Paal, Belgium) according to a protocol previously described [29]. Quantitative analysis of the locomotion was obtained for each walking cycle by comparing the percentage of the total length (100%) of the stance phase (which takes up 62% of the full gait cycle) to (1) the initial period (reception of the foot) from the heel strike (initial contact) to the foot flat (loading response), (2) the intermediate period (foot flat) from the foot flat to the heel off (terminal stance), and (3) the last period (the push) from the heel off to the toe off (preswing) [32].

*Stabilometry*. Postural oscillations were recorded on a stabilometric platform (AFP/APE85 40 Hz /16-bit, Fusyo-Medicapteur®, Dekra certification, Balma, France) and analyzed with the software (V.1.2.1, Fusyo-Medicapteur®) coupled to the platform. The posturographic platform measured 530 mm × 460 mm × 35 mm and was equipped with three pressure gauges (hysteresis <0.2%). In accordance with recommendations from the French Posturology Association and the International Society for Posture and Gait Research (ISPGR), patients were positioned standing on the platform, lowered arms, barefoot, with heels 2 cm apart and feet away at 30° so that the centroid of sustentation polygon was located on the sagittal axis of the platform at a known distance back from the electrical center of the platform. As previously described [29], with this standardized position, a first recording was made with open eyes focused on a point marked at a distance of one meter and a second with closed eyes to determine the part of the visual input in the postural control. Measured variables were (1) the statokinesigram surface, (2) X and Y means (Xm and Ym) defined as mean placement of center of pressure in the frontal (X) and sagittal (Y) planes, (3) length/surface (LFS) of the statokinesigram that measures the path of the center of pressure per unit area, and (4) variance of Y (VFY).

### 2.6. Electrophysiological Recordings

In accordance with the SENIAM international recommendations (Surface ElectroMyoGraphy for the Non-Invasive Assessment of Muscles, 1999) and in order to maintain the interelectrode impedance below 5 kΩ, the skin was carefully shaved, slightly roughed, degreased, and disinfected by using alcohol wipes. If the interelectrode impedance was higher than 5 kΩ, the skin preparation steps were repeated. Surface bipolar electrodes (Universal ECG Ag/AgCl, Control-Graphique SA, Brie-Comte-Robert, France) were placed on the* soleus* (SOL), the* tibialis anterior* (TA), the* biceps femoris* (BF), and the* vastus medialis* (VM) muscles of the two lower limbs. The interelectrode distance was set at 25 mm center to center. For SOL, the electrodes were positioned along the midline of the leg, about 50 mm below* gastrocnemii* insertion of Achilles tendon. For TA, the electrodes were placed on the line of the fibula to the distance of 1/3 from the end of the medial* malleolus*. For BF, the electrodes were placed at 50% of the line formed between the ischial tuberosity and the lateral epicondyle of the tibia. For the VM, the electrodes were fixed to the distance of 2/3 from the line originating at the anterior superior iliac and up to the medial face of the patella. The four reference electrodes were positioned on the patella or on the ankle. Each electrode was attached firmly to the skin using adhesive strips. The position of each electrode was carefully measured, recorded in a notebook, and photographed to ensure identical positioning of the electrodes during the following sessions. The electromyogram (EMG) activities of these muscles were recorded on dynamic (locomotion over a distance of 10 meters) condition and on static (standing) condition either with eyes opened or with eyes closed. The signals were amplified (x10000), filtered (bandwidth 10-500 Hz), sent to a computer via an analog-digital converter interface (Biopac® MP100), and analyzed with AcqKnowledge® 3.7.3 software (Biopac System Inc.). Root Mean Square (RMS)-EMG (expressed in *μ*V) of each muscle was calculated for each burst and averaged during walking for each limb (pain and control limb) or calculated within 5 periods of 10 s for static condition.

Then, the subject was comfortably seated on a home-made ergonomic chair in order to record muscle reflexes. Recording electrodes were left on the 4 muscles. In patients with plantar* fasciitis*, the pain limb was blocked with a knee and ankle angle set at 90°. In healthy participants, the dominant limb was blocked in the same position. This position minimized* gastrocnemii* muscle contribution to the force production [33] ensuring participation of more than 80% of the* soleus* muscle [34]. The limb position was maintained with a strap on the foot and the knee and by a plate equipped with protective foam above the knee, in order to prevent lifting of the heel during the* soleus* muscle contraction. To reduce movement of the upper body during contractions and therefore to reduce the contribution of the thigh extensor muscles and hip flexor muscles, a belt was fixed to the hip of the subject. The contralateral limb was kept the most relaxed. Particular attention was focused on the head position in order to prevent its rotation during the recordings because any movements lead to vestibular influences on motor reflexes [35]. The foot was placed on a base related to a strain gauge platform (Tedea-Huntleigh Load Cell® S-type 615, VPG Transducers, Vishay Precision Group, Inc., PA, USA) that recorded the force developed during the* soleus* muscle contraction. The signal obtained from the strain gauge was sent to a computer via an analog-digital converter (sampled at 2000 Hz, filtered with Low Pass at 150 Hz) interface (Biopac® MP100, Biopac System Inc., CA, USA) and analyzed with AcqKnowledge® 3.7.3 software (Biopac System Inc.). All neuromuscular tests were therefore carried out under isometric conditions with the same position on the chair for each subject.

In order to record the M(muscle)-, H(reflex)-, and V(volitional)-waves on the SOL muscle, the* tibial* nerve was stimulated using a nerve stimulator (Constant Current Stimulator, Model DS7A, Digitimer® Ltd., Hertfordshire, England) via a self-adhesive electrode (Universal ECG Ag/AgCl, Control-Graphique SA) placed in the popliteal fossa. The anode (self-adhesive electrode 50 x 89 mm, Dura-Stick®, DJO-Global LLC, CA, USA) was positioned on the anterior surface of the knee. Single and biphasic pulses with 1 ms duration and variable intensity (20 mA to 160 mA) for a maximum voltage of 400 V were delivered. Before any prior recordings, the optimal stimulation site was localized with a tungsten electrode (5 mm diameter). It corresponded to the skin position where the largest H-wave amplitude was obtained for a current set at 20 mA intensity. In order to minimize intraindividual variability, the stimulation site was localized in the same way for the following recording sessions (W3, W6, and W9). Indeed, it was essential to standardize. The electrode position was determined in the standing position. Once the site was found, the stimulating electrode was placed and firmly secured by a strap.

**Figure 2:**
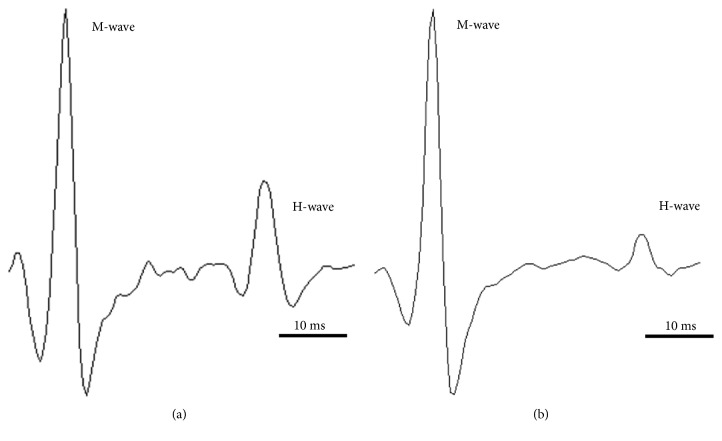
Examples of waves recordings. (a) In a healthy participant. (b) In a patient with a plantar* fasciitis*.

Transcutaneous stimulation of the* tibial* nerve produced two EMG responses (Figure 2). The earlier response (< 10 ms latency), the M-wave, was due to direct activation of the motor axons in the tibial nerve and did not involve a spinal circuit. The second response (> 30 ms latency), the monosynaptic H-reflex, was due to the activation of muscle afferents in the tibial nerve that synapse on spinal motoneurons. Thus, reflex was recorded at rest (H-reflex) and during voluntary contraction (H_sup_ and V-wave). H-reflex recording allows evaluation of the proportion of motoneurons activated [36] and the transmission between muscle afferents and motoneurons [37]. It is a monosynaptic reflex bypassing the muscle spindles, artificially evoked by electrical stimulation applied to a peripheral nerve. Briefly, the amplitude of the H-reflex depends on the recruitment of motor unit originating from Ia afferents activation [38]. Regarding H_sup_ and V-wave, when a muscle is relaxed (at rest), supramaximal stimulation of the motor fibers provides a maximal M-wave (M_max_, amplitude represents an index of sarcolemma excitability) that abolishes the H-wave. However, when the same stimulation is evoked during a voluntary contraction, the M-wave is followed by a wave appearing with a delay similar to the H-wave delay. Indeed, during a voluntary contraction, electrical stimulation of a mixed peripheral nerve depolarized afferent and efferent axons. On the efferent side, evoked nerve influxes are propagated orthodromically to the muscle where they generated with the motor descending voluntary influxes a M-wave (M_sup_) and antidromically to the spinal cord. Descending influxes by the corticospinal and extrapyramidal pathways collide with the antidromic evoked influxes [39]. The probability of this antidromic collision rises with increasing voluntary muscle activation. Thus, when the muscle is sufficiently activated, the cancellation of the wave by antidromic impulses from descending pathways allows influxes conveyed orthodromically by afferent fibers to reach the muscle [40]. Under these conditions, it is possible to measure a variant of the H-reflex called H_sup_ when voluntary contraction reaches 60% of the maximal voluntary contraction and V-wave (volitional wave or volley of H-reflex impulses) when the contraction is maximal. H_sup_ and V waves allow evaluating the modulation of the spinal loop and reflect the efficiency of the transmission between Ia afferent and *α*-motoneuron during voluntary contraction, i.e., the presynaptic inhibition [35] and the level of afferent and descending neural drive [39].

Measurements started by determining the H and M recruitment curves. To achieve that, the intensity of stimulation (starting at 20 mA) was gradually increased every two stimulations until the maximal H-reflex (H_max_) was reached. H_max_ was detected when the H-reflex began to decrease significantly while the M-wave increased. For a better reproducibility, many precautions have been taken during recording. Stimulations were separated by an interval of at least 10 seconds [38] and performed at the end of the expiration time. The subject was also asked to stand still (no movement of the head or hands), eyes closed and completely relaxed in a quiet environment (for this purpose, the patient had to wear hearing protection). If the subject was disturbed or if he moved, calm recovery was expected before further stimulation. When the maximal amplitude was found, 3 to 5 stimuli at the same intensity were performed to average the results. The corresponding M-wave (M_Hmax_) was also noted. The peak to peak amplitudes of H_max_ and M_Hmax_ were measured. The results of previous sessions were viewed to ensure the consistency of measures. Once H_max_ value was obtained, the stimulation intensity was increased until the disappearance of the H-reflex and M-wave stabilization (maximum intensity). Then, 3 to 5 pulses were delivered at supramaximal intensity (125% of the maximum intensity) to obtain the maximal M-wave (M_max_).

Afterwards, the subject was encouraged to lift the heel by contracting solely the SOL without other muscles contribution. Surface electrodes from the TA, BF, and VM muscles were used to ensure that their participation in the force production was negligible. The subject was asked to keep his arms crossed over the chest during contractions. During training, the subject visualized the EMG of the 3 muscles and the force developed on the strain gauge platform. Then, the feedback was removed and he was asked to perform 3 maximum voluntary isometric contractions (MVC) using only the SOL. During each MVC, the subject was verbally encouraged and the maximal forces developed were noted. Because it was previously shown that the H-reflex increased linearly with force production until reaching a plateau at 40-60% of the maximal voluntary force [41], the subject was asked to produce a force corresponding to 60% of MVC (H-reflex amplitude being maximal at this force level). An electrical pulse corresponding to the intensity of H_max_ was delivered during a stable period of contraction. The superimposed H-reflex (H_sup_) and the corresponding M-wave (M_Hsup_) were recorded on 3-5 trials. Then, the patient performed 3 MVC stabilized for 3 seconds during which a pulse of supramaximal intensity was delivered. The wave with a similar delay (between 30 and 45 ms) to the H-wave corresponded to the V-wave [41]. The corresponding M-wave was also recorded (M_sup_).

Measurements were randomized. All wave or force amplitudes were measured peak to peak. Inconsistent results were systematically excluded from the study. Amplitudes of SOL H_max_, M_Hmax_, and M_max_ were measured at rest. H_max_/M_max_ and M_Hmax_/M_max_ ratios were thus determined. During voluntary contractions, amplitudes of H_sup_ and M_Hsup_ (measured at intensity used to evoke H_max_) and V as M_sup_ (during a maximum voluntary isometric contraction) were recorded. Thus, H_sup_/M_sup_, V/M_sup_, and MH_sup_/M_sup_ ratios were determined.

H_max_/M_max_, H_sup_/M_sup_, and V/M_sup_ ratios were calculated to ensure that any changes in the evoked H_max_, H_sup_ and V amplitudes were not due to changes at the muscle fiber membrane or neuromuscular junction [42]. To ensure that the same proportion of *α*-motoneurones was activated by the electrical stimuli, the M_Hmax_/M_max_ ratio was compared to the M_Hsup_/M_sup_ ratio [43].

### 2.7. Statistical Analysis

Primary dependent variables were RMS, H_max_, M_max_, H_sup_, M_sup_, M_Hmax_, M_Hsup_, and V waves. Secondary dependent variables were pain level, parts of the stance phase and stabilometric parameters (stance surface, Xm, Ym, LFS and VFY). A power analysis was used to determine the number of patients to include in the protocol. The calculation of the sample size was performed in a pilot study involving 6 subjects (3 healthy participants and 3 patients with plantar* fasciitis*), in which there was a H_max_/M_max_ ratio lower than 0.167 in the suffering patients compared to the healthy participants. Using a standard deviation of 0.08, *α* = 0.05 and 90% power, the sample was estimated as 10 subjects (5 in each group).

Normality of the data was checked and subsequently confirmed using the Kolmogorov-Smirnov test. Other assumptions (independence of cases, homoscedasticity) were also checked. Differences were tested by analysis of variance (ANOVA test, time effect) completed by a Tukey* post hoc *test. Data processing was performed using commercially available statistical software (GraphPad Instat® 3.00, GraphPad Software, Inc., San Diego, CA, USA). Data were expressed as mean±SEM. Results were considered statistically significant if the p-value fell below 0.05.

Finally, in order to facilitate the interpretation of the results and to evaluate the clinical relevance, according to Cohen (1988)[44], for pain perception at the end of the protocol (W9), the effect sizes were calculated by dividing the difference between mean values (preintervention scores* vs.* postintervention scores) by the pooled standard deviation.

## 3. Results

Analyses were focused on 5 healthy participants and only on the 10 patients presenting plantar pain who participated to the nine weeks of study. No participant reported adverse effects from wearing foot orthotic devices.

### 3.1. Pain

**Figure 3:**
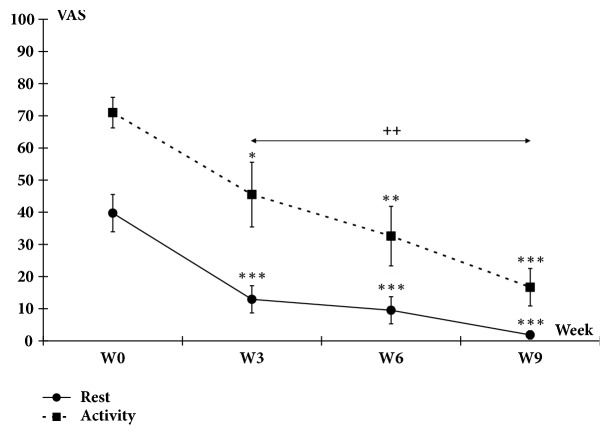
Plantar pain assessment. The intensity of pain experienced at rest and during activity by the patient was subjective. It was assessed on an analog visual scale (AVS) graduated from 0 (no pain) to 100% (‘worst pain imaginable'). Significant differences with W0 are indicated by asterisks (*∗*: p<0.05; *∗∗*: p<0.01; *∗∗∗*: p<0.001). Differences between W3 and W9 are indicated by crosses (++: p<0.01). Data are mean±SEM.

In the Control group, no plantar pain was reported by all the participants throughout the experimental sessions. At rest, the mean pain score was significantly reduced from the third week after the beginning of the treatment (W3: -67.5%, p<0001; W6: -75.01%, p<0.001; and W9: -95.31%, p<0.001) when compared to W0. During activity, reported mean pain score was also significantly reduced from the third week (W3: -35.91%, p<0.05; W6: -53.05%, p<0.01; and W9: -76.23%, p<0.001) when compared to W0. Furthermore, statistical analysis indicated a significant (p<0.01) decrease from W3 to W9. However, although at rest the pain almost disappeared (1.87±1.31%), low persistent pain (16.87±6.19%) was reported during the activity after 9 weeks of treatment (Figure 3).

Considering the mean values obtained at preintervention and postintervention, the effect sizes were* r*=0.804 (Cohen's* d*=2.70) and* r*=0.841 (Cohen's* d*=3.11) for rest and during exercise, respectively.

### 3.2. Locomotion

The dynamic analysis of the locomotion was assessed by measuring the percentage of the 3 steps of the stance phase of the gait cycle. Although the first and the third steps seemed to increase and the second step seemed to decrease from W1 to W9, repeated ANOVA analysis does not reveal significant changes in the 3 steps of the stance phase over time. However, when compared to healthy participants, data indicated that (1) the first step (from the initial contact to the loading response) of the stance phase was significantly (p<0.001) lower during the nine weeks of experiment (the normal value being 25% of the stance phase), (2) the second step (from the loading response to the terminal stance) was significantly higher (p<0.01) only during the third week (from W1 to W3) and then returned to normal values (45% of the stance phase), and (3) the last step (from the terminal stance to preswing) was significantly higher (p<0.05) from W6 to W9 than normal value (30% of the stance phase)(Table 2).

### 3.3. Stabilometry

**Figure 4:**
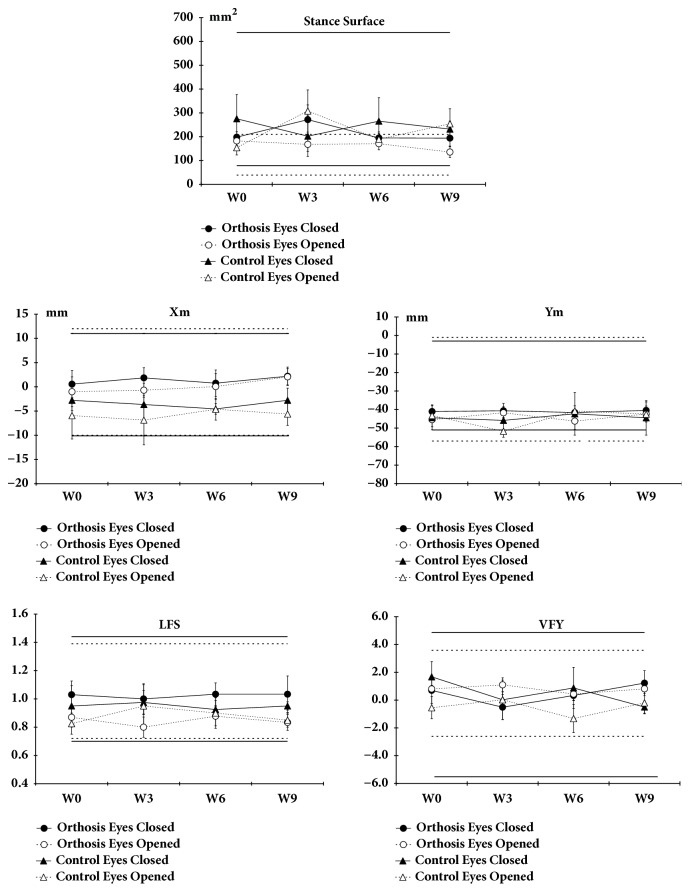
Stabilometric parameters. Five selected parameters (Stance surface, Xm, Ym, LFS and VFY) were analyzed at W0, W3, W6 and W9. The solid lines represent lower and upper limits for subjects with eyes closed. Dotted lines represent lower and upper limits for subjects with eyes opened. Data are mean±SEM.

In all groups, all measured static stabilometry parameters remained relatively stable throughout the experimental sessions and nothing indicated that the patients were imbalanced despite a plantar* fasciitis*. Stance surface, Xm, Ym, LFS, and VFY parameters analysis indicated that our suffering patients exhibited values within the lower and upper limits when eyes were opened or closed and no difference was found when compared to Control group. Furthermore, there was no significant change in the parameter from W0 to W9 (Figure 4).

### 3.4. EMG Recordings

**Figure 5:**
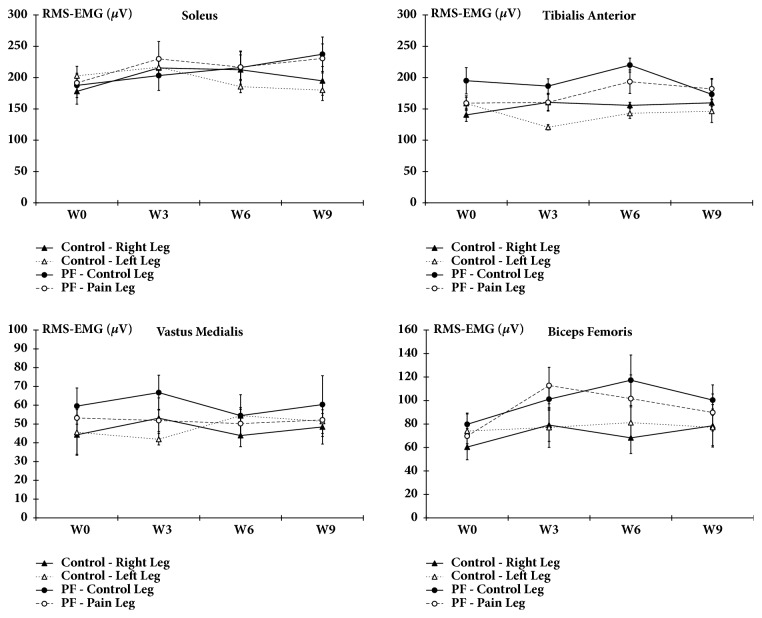
Electromyographic recordings. During walking, RMS-EMG of* soleus*,* tibialis anterior*,* vastus medialis *and* biceps femoris* was recorded at W0, W3, W6 and W9. Data are mean±SEM.

In all subjects (healthy participants and patients suffering from plantar pain), RMS-EMG remained fairly stable during the nine weeks of the protocol either during walking or in standing position. During walking, no RMS-EMG change was found between the control and the painful limb in patients suffering from plantar* fasciitis* over time. Furthermore, no difference was found between the Control group and the group of patients during the nine weeks of the protocol (Figure 5). Finally, during static (standing) position and when eyes were closed or opened, no RMS-EMG difference was found either between the two lower limbs of patients suffering of plantar* fasciitis* or between the healthy participants and the group of patients regardless of the session (*results not shown*).

### 3.5. Reflex Recordings

**Figure 6:**
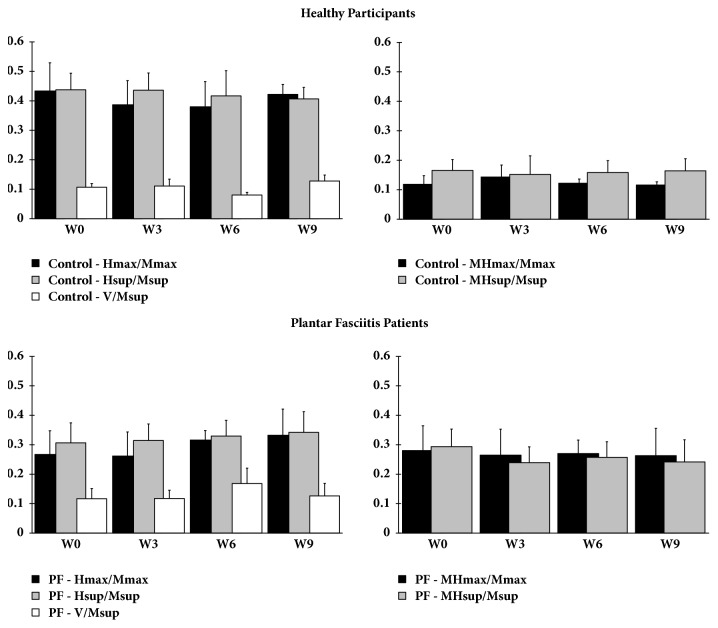
Spinal reflex activity. H and M-waves were recorded at rest and during* soleus* muscle contraction and H_max_/M_max_, H_sup_/M_sup_, V/M_sup_, M_Hmax_/M_max_, M_Hsup_/M_sup_ ratios were calculated at W0, W3, W6 and W9. Data are mean±SEM.

In all subjects (healthy participants and patients suffering from plantar pain), H_max_/M_max_, H_sup_/M_sup_, and V/M_sup_ ratios remained stable during the nine weeks of the protocol. Furthermore, M_Hmax_/M_max_ and M_Hsup_/M_sup_ ratios did not change between each experimental session indicating that the measurement conditions of motor reflexes were stable and reproducible. Finally, our results revealed that the H_max_/M_max_ and H_sup_/M_sup_ ratios were significantly higher (p<0.01) and that M_Hmax_/M_max_ and M_Hsup_/M_sup_ were significantly lower (p<0.01) in healthy participants compared to patients with plantar* fasciitis*. Our results also indicated no difference in the V/M_sup_ ratio between groups (Figure 6).

## 4. Discussion

Our study aimed to determine if reduction of pain in patients with plantar* fasciitis* with a conservative treatment based on plantar orthoses known to correct postural deviations and muscle deficiencies and to address specific imbalances during foot placement or gait was related to changes in reflexes and muscle activity of the muscles of the lower limbs.

Our results indicated that the H_max_/M_max_, H_sup_/M_sup_, and V/M_sup_ ratios remained stable during the 9 weeks of the protocol indicating that pain decrease at rest and during activity reported by patients wearing orthoses is not related to change in neural strategy. We also noted that H_max_/M_max_ ratio was higher and M_Hmax_/M_max_ ratio was lower in healthy participants compared to patients with plantar* fasciitis* indicating that in this last group the number of *α*-motoneurons directly activated was greater and the number of *α*-motoneurons reflexively activated was lower which makes it possible to assume with caution that (1) presynaptic inhibition was greater and/or (2) Ia afferents were more difficult to activate and/or (3) threshold of excitation was lower in *α*-motoneurons. We also noted the same results during voluntary contraction, i.e., H_sup_/M_sup_ ratio was higher and M_Hsup_/M_sup_ was lower in healthy patients. We also observed no change in RMS-EMG from* vastus medialis* (VM),* tibialis anterior* (TA),* soleus*, and* biceps femoris* (BF) muscles during walking and static position between the two limbs of patients suffering from plantar* fasciitis* and when compared to healthy participants. Furthermore, although values of the 3 steps of the stance phase of the gait cycle were significantly different from values of healthy participants, no significant change in the stance phase was observed during the 9 weeks of the protocol. Finally, stabilometric parameters indicated no change in the postural tone after wearing the plantar orthoses.

### 4.1. Pain

In our study, plantar orthoses allowed a significant pain regression throughout our protocol at rest and during activity. Although pain did not completely disappear during activity as already observed in other studies [19], the quality of life of patients was improved during daily or sport activities indicating the beneficial effect of the foot orthoses based on sound biomechanical principles that are designed to correct postural deviations and muscle deficiencies and to address specific imbalances during foot placement or gait. Although the mechanical correction is the main mechanism involved with these orthoses, a proprioceptive stimulation of the sole of the foot is also present to a lesser extent [27]. Together, mechanical correction and proprioceptive stimulation could be the cause of pain reduction.

### 4.2. Locomotion

Despite pain decrease during the 9 weeks of the protocol, the dynamic analysis of the locomotion assessed by measuring the percentage of the 3 steps of the stance phase of the gait cycle indicated that the locomotion in the group of patients suffering from plantar* fasciitis* remained different from the Control group and unchanged from W0 to W9. This pathological gait pattern persisted despite correction of the biomechanical function of the foot and support of the arch of the foot. Thus, due to its implication and solicitation in the different phases of the stance phase of the gait cycle, any plantar fascia alterations (stiffness, etc.) may alter the gait cycle. Furthermore, because the sole of the foot plays a major role in regulating balance and posture control during standing and walking [45], plantar fascia pain may induce changes in the functioning of the sensorimotor loop and in the activity of the muscles of the lower limbs and thus alters the gait cycle. Conversely, we can not exclude that changes in locomotion are a strategy adopted by the subject to reduce pain.

The literature indicates that there is little consensus regarding the effect of plantar* fasciitis* and poor information on the effect of foot orthoses on the gait cycle. Previous studies indicated that plantar pain induced changes in foot roll-over patterns, thus causing load reductions in the rearfoot and load increases in other plantar regions, such as the midfoot, possibly owing to the protective mechanisms of pain [31]. Kelly et al. reported no difference in the plantar pressure patterns among nonathletic individuals with or without plantar* fasciitis* during gait [46]. It was also reported that the adaptation of the foot could result in lowering of the arch or its elevation [47]. Liddle et al. observed no differences between symptomatic and asymptomatic limbs with respect to the magnitude and timing of the vertical heel strike transient or first vertical force maximum in patients walking at their preferred speed [48] contrary to Katoh et al. that showed a substantial change in the ground force reaction [49]. Wearing et al. reported gait adjustments that result in reduced force beneath the rearfoot and forefoot of the pain foot and an increase in digital loading suggesting that digital function plays a protective role [50]. Furthermore, Prichasuk and Sbhadrabandhu reported that the calcaneal lateral tilt angles were decreased in symptomatic patients when compared to control group [51]. A decrease in the ankle dorsiflexion range of motion is also described in individuals with plantar* fasciitis* associated with less flexibility of the* triceps surae* muscular group and decreases in extension of the toes are described [6, 52]. Finally, Lin et al. reported that anterior rocker sole shoe reduced better than flat insoles the windlass effect occurring during the preswing phase of gait cycle in which the peak tensile and force of the plantar fascia are reached [53]. The authors concluded that such footwear that reduces the tensile strain and force on the plantar aponeurosis is appropriated for treating foot disorders such as plantar* fasciitis*.

In our study, because we did not observe a return to a normal locomotion in patients with plantar* fasciitis* despite a decrease of pain, we can assume that the plantar aponeurosis had not recovered 9 weeks after wearing biomechanical/functional orthotics and that the reduction of pain may be a result either of change in the distribution of plantar loads or of other processes such as neural adaptations implemented to block the transmission of painful influxes.

### 4.3. Postural Regulation

In our patients with plantar* fasciitis*, statokinesigram surface, Xm, Ym, LFS, and VFY parameters remained, as in healthy participants, within the lower and upper limits before (W0) and after (W1 to W9) wearing foot orthoses. These results indicate that the postural control is not compromised despite pain. A change in postural control could have been expected over time to explain the reduction of pain. However, despite wearing biomechanical/functional orthoses capable of controlling functional pathology of the foot by maintaining the foot in its neutral position or close to it, or to change plantar sensitivity as suggested by Vie et al. [54], we do not observe any postural change from W1 to W9 that could explain the reduction of pain. To our knowledge, no study measured stabilometric parameters in patients with plantar* fasciitis* and reported if foot orthoses change the postural control over time. However, the literature reported that patients with plantar* fasciitis* presented a slight limitation of dorsiflexion of the hallux that was not present in healthy participants and that hallux dorsiflexion and the Foot Posture Index were inversely correlated [55]. Another study reported that runners with symptoms or histories of plantar* fasciitis* did not differ in rearfoot valgus misalignments but showed increases in the longitudinal plantar arch during bipedal static stance, regardless of the presence of pain symptoms [56]. The same authors reported that the patterns of plantar pressure distribution were not affected in recreational runners with plantar* fasciitis* when compared to runners without the disease.

Molded foot orthoses are known to increase stability of the foot, to reduce plantar fascia strain, and to change frontal plane and rearfoot alignment. Wearing foot orthotics improves, by mechanical effect, the postural control by optimizing positioning of the foot [57–59]. Furthermore, the stresses exerted on the plantar aponeurosis when standing, walking, running, or jumping are diminished which may explain the reduction of pain when wearing foot orthoses during daily and sport activities.

In light of our findings, we can conclude that the reduction of pain results from a temporary reduction in stresses on plantar fascia and not from a long-term modification of the postural control, i.e., protection of the plantar fascia occurs only when the patient wore his orthoses and this effect may last several hours, days, or weeks after removing the orthoses. It is likely that if the subjects stop wearing their foot orthoses, the pain will reappear after a few hours, days, or weeks.

### 4.4. EMG

If the literature reports that foot orthoses affect frequency components of muscle activity in the lower extremity during running or walking in subjects without the disease [60, 61] or with chronic ankle instability [62], few findings were devoted to record electromyographic parameters of limb and foot in individuals with plantar* fasciitis*. Cheung et al. described that intense muscle contraction of the plantar flexor muscles increases the risk of developing symptom of pain related to the disease [63]. It was also shown that alteration or impairment of plantar foot intrinsic muscles by blocking the tibial nerve [64] or by fatiguing plantar muscle [65] influences the height of the navicular and shape of the medial longitudinal arch. Other authors suggested that* posterior tibialis* muscle that provided the most significant dynamic arch support during the stance phase of the gait can be easily fatigued because of its lengthened position necessary to control excess motion [66]. Kibler et al. reported that individuals with plantar* fasciitis* present less flexibility of the* triceps surae* (*gastrocnemius* and SOL) muscular groups [67]. Thus, in patients with plantar* fasciitis*, elevation of the heel increases flexibility of the Achille tendon and consequently decreases the tension of the* gastrocnemius* muscle. This result can be obtained with orthotic devices elevating the heel. Finally, other distal muscles such as* flexor digitorum longus*,* flexor hallucis brevis*, and* peroneus longus* and proximal muscles such as* gluteus medius*,* gluteus minimus*,* tensor fasciae latae*, and quadriceps were described to be involved in windlass mechanism and plantar fascia abnormalities, respectively [68–70].

In our study, we did not observe any EMG changes in the SOL, TA, BF, and VM muscles of the two lower limbs during locomotion and static conditions in patients with plantar* fasciitis*. We also did not observe any differences between patients suffering from plantar* fasciitis* and healthy subjects. These results indicate that plantar* fasciitis *does not affect these muscles. In the leg, the SOL muscle mainly acts to flex the foot and to support the knee while the TA is responsible for dorsiflexing and inverting the foot. In the thigh, the BF performs knee flexion while VM is involved in the knee extension. Our results are in accordance with the absence of changes in postural regulation and suggest that these muscles are not involved in the altered locomotion in patients with plantar* fasciitis*.

### 4.5. Reflexes

Our study showed that, in healthy participants as in suffering patients, the value of the M_Hmax_/M_max_ ratio was stable regardless of the session indicating that the same proportion of *α*-motoneurons was activated from W0 to W9. Thus, we did not observe any changes in spinal reflexes over time despite pain reduction suggesting that no neural strategy was involved in reduction of pain. It was already shown that stretching or counter resistance movement in patients with plantar* fasciitis* did not modify H-reflex as well as stretch reflex despite a short term decrease in pain sensation [71].

Furthermore, in healthy participants the H_max_/M_max_ ratio was higher than that of patients with plantar* fasciitis* indicating that a greater pool of *α*-motoneurons was activated by the Ia afferents and/or a greater motoneuron excitability. However, M_Hmax_/M_max_ ratios were always lower in healthy participants compared to patients with plantar* fasciitis* indicating that the fraction of *α*-motoneurons directly activated when H_max_ is obtained is lower in healthy participants compared to patients with plantar* fasciitis*. Because M_max_ was identical in both H_max_/M_max_ and M_Hmax_/M_max_ ratios, we can conclude that mean amplitude of H_max_ was always greater than that of M_Hmax_. This means that, at the intensity of stimulation to obtain H_max_, the number of *α*-motoneurons (mainly slow motoneurons) transynaptically activated was higher than the fraction of *α*-motoneurons (both slow and fast) directly activated.

Because H_max_/M_max_ ratio was higher and M_Hmax_/M_max_ ratio was lower in healthy participants compared to patients with plantar* fasciitis*, we could conclude that in this last group the number of *α*-motoneurons directly activated was greater and the number of *α*-motoneurons reflexively activated was lower suggesting that (1) presynaptic inhibition was greater and/or (2) Ia afferents were more difficult to activate and/or (3) threshold of excitation was lower in *α*-motoneurons. However, it is difficult to compare H_max_/M_max_ and M_Hmax_/M_max_ ratios together and to propose a reliable explanation because the different categories of *α*-motoneurons are not activated similarly by the two ways (transynaptically and directly).

Pain may have several origins including inflammation of the plantar fascia [12, 72]. Indeed, it was reported that chronic inflammation is the secondary mechanism leading to degenerative changes of the aponeurotic tissues after mechanical overload and excessive stretching [31, 73]. The literature reports that inflammatory mediators released during exercise or injected in the muscle activate thin joint or muscle afferent fibers from groups III and IV (and nociceptive fibers) known to regulate the central drive and spinal reflexes [74–76]. Indeed, these afferents are described to modulate the sensorimotor loop by inhibiting Ia afferent fiber, by modulating spindle sensitivity and motoneurons activity, and to project toward the cortical motor area [75, 77]. Consequently, if reduction of pain in patients wearing foot orthoses was associated with decrease in inflammation, a change in spinal reflexes should be observed over time. Because we did not observe any change in H_max_/M_max_, H_sup_/M_sup_, and V/M_sup_ ratios during the nine weeks of the protocol, we can conclude that reduction of pain in patients wearing plantar orthoses was not associated with change in reflexes or consequently with the reduction of inflammation, i.e., the spinal motoneuron responsiveness was not affected and voluntary motor output (motoneuron recruitment and/or firing frequency) was not increased despite wearing plantar orthoses. Thus, our results suggest that either the level of inflammation remained constant during the nine weeks of the protocol despite wearing orthoses or no inflammation was present in patients with plantar* fasciitis* during the experimental session. However, comparison between the Control group and patients with plantar* fasciitis* revealed that the H_max_/M_max_ and H_sup_/M_sup_ ratios were significantly higher in healthy participants indicating that spinal reflexivity was different in the two groups. Our results also indicate no difference in the V/M_sup_ ratio between groups. The H_max_/M_max_ is related to the type of physical training (endurance* vs.* resistance) and to the level of training [78]. Because it was shown that this ratio was higher in athletes than in sedentary subjects [78], the lower ratio we observed in patient with plantar* fasciitis* may be the consequence of the decrease activity in patients with plantar pain. As for H_max_/M_max_ ratio, the H_sup_/M_sup_ ratio, obtained during MVC, is considered as a global index of the spinal modulation produced by presynaptic inhibition, motoneuron excitability, collision in antidromically activated axons, and Renshaw cell inhibition. Thus, our results may suggest that, during MVC, motoneuron pool excitability was affected in group of patients with plantar* fasciitis*. Reduction of activity in patients with plantar* fasciitis* may have affected the recruitment and/or rate coding of the motoneurons activated by the H-reflex afferent volley. Thus, as previously suggested [79], change in muscle phenotype may affect the calculated ratios. Because V/M_sup_ ratios were similar between groups, we can conclude that the descending drive from higher centers was similar [39]. Finally, we observed that, during the 9 weeks of the protocol, the M_Hsup_/M_sup_ ratios were higher in patients with plantar* fasciitis* compared to healthy participants indicating that the proportion of *α*-motoneurons directly activated by the electrical stimuli was different in the two groups, the group of patients with plantar* fasciitis* presenting a higher proportion of motor unit directly activated [43].

Whatever the origin of pain (inflammation or collagen degeneration without inflammation) [73], our results indicate that the spinal sensorimotor loop remained affected in patients with plantar* fasciitis* during the nine weeks of the protocol despite wearing orthoses and that the reduction of pain was not due to neural changes or healing but probably due to an unloading effect on painful plantar zone.

### 4.6. Limitations

As for the majority of experiments involving humans, despite the drastic criteria of inclusion and exclusion of subjects, some limitations in this study can be pointed. First of all, there is some intragroup variability between subjects in terms of age, weight, anthropometric parameters, level of daily activity, and perception of pain. Some intergroup differences were also present, the suffering patients (51.0±3.5 years) being older than healthy participants (30.6±2.1 years). Secondly, because enrolled patients presented chronic plantar pain for at least 6 months, some differences may exist between a patient suffering for 6 months and another suffering for a year or more; with time, neural and morphological adaptations can take place. Third, the origin of plantar pain may have different origins (inflammation or tissue degeneration followed by an inflammation). It is important to keep in mind that there is heterogeneity between patients despite all our efforts in patient selection and that these differences can diminish the power of our results. Furthermore, because muscle recordings were performed extracellularly, some heterogeneity can also be introduced despite the fact that we had taken care to replace the electrodes always in the same place during the different experimental sessions and took the necessary precautions so that the conditions of recording of the signals were always the same. Only an animal model of plantar* fasciitis* would further reduce this heterogeneity, but to our knowledge this model does not exist. Finally, despite a pain reduction with the use of orthotics, it is difficult to distinguish between patients who recover spontaneously and those who respond to such conservative intervention. For this later parameter (which is finally the only one interesting health care clinicians and patients receiving care), because statistical significance is based on hypothesis testing that gives enough evidence against the null hypothesis (i.e., there is no difference between groups or independent variable does not have an effect on the dependent variable) or not and because it was possible to have statistical significance without having clinical relevance, we calculated the effect size which simplifies the transfer of knowledge from research to practice [80]. Thus, in our experiments, the calculation performed at rest and during exercise indicated large effect sizes (pre-* vs.* postintervention) at W9 confirming the effectiveness of biomechanical orthoses.

### 4.7. Conclusions

Wearing plantar orthoses based on sound biomechanical principles is an efficient conservative strategy to reduce pain in patients with plantar* fasciitis*. However, during the 9 weeks of the protocol, patient relief is not associated with restoration of locomotion and spinal reflexes. No neural changes seem to occur despite pain decrease. It is possible that the study duration was not long enough to observe the expected changes. If the reduction of pain is simply due to the change in pressures on the painful plantar areas that induce a decrease of stretch and tear on the plantar fascia ligaments, it is likely that a return to physical activity too early will lead to a return of pain. Our results suggest that, after 9 weeks of wearing plantar orthoses based on sound biomedical principles, patients are not yet cured and return to physical activity should be delayed. It could be interesting to compare our results with those obtained with orthoses mainly based on proprioceptive stimulations in order to determine which type of orthotic devices is most effective.

## Figures and Tables

**Table 1:** Anthropometric characteristics.

**Patients**	**Age** **(year)**	**Weight** **(kg)**	**Size** **(cm)**	**Affected Leg **	**Pain Perception (**%**)**	**Sex**
Patient n°01	25	68	161	L	60	F

Patient n°02	51	81	180	R	60	M

Patient n°03	45	72	170	L	90	M

Patient n°04	57	72	171	L	90	M

Patient n°05	57	84	173	L	60	M

Patient n°06	58	77	171	R	60	M

Patient n°07	63	62	168	L	90	F

Patient n°08	44	84	170	L	60	F

Patient n°09	60	76	174	L	60	M

Patient n°10	50	84	181	L	80	M

**Mean**	**51.0**	**76.0**	**171.9**	**2 R / 8 L**	**71**	**3 F / 7 M**

**SEM**	**3.5**	**2.3**	**1.8**		**4.5**	

Control n°01	**28**	**51**	**155**	NA	0	F

Control n°02	**35**	**55**	**165**	NA	0	F

Control n°03	**25**	**58**	**169**	NA	0	F

Control n°04	**29**	**71**	**175**	NA	0	M

Control n°05	**36**	**65**	**171**	NA	0	M

**Mean**	**30.6**	**60.0**	**167.0**		**0**	**3 F / 2 M**

**SEM**	**2.1**	**3.5**	**3.4**		**0**	

	**p<0.05**	**p<0.01**	**NS**		**p<0.001**	**NS**

Abbreviations: F, female; L, left; M, male; NA, not applicable; NS, not significant; R, right; SEM, standard error of the mean. Differences between groups were indicated on the last line of the table.

**Table 2:** *Quantitative analysis of the 3 steps of the stance phase*. **Step 1:** the initial period (reception of the foot) from the heel strike (initial contact) to the foot flat (loading response) representing around 25% of the stance phase in normal cycle. **Step 2:** the intermediate period (foot flat) from the foot flat to the heel off (terminal stance) representing around 45% of the stance phase in normal cycle. **Step 3:** the last period (the push) from the heel off to the toe off (preswing) representing 30% of the stance phase in the normal cycle. Compared to respective standards: *∗∗∗*, p<0.001; ++, p<0.01; *δ*, p<0.05.

	**W1**	**W3**	**W6**	**W9**
**Healthy participants**				

**Step ** **1**	26.33±2.03	24.12±1.95	23.98±1.25	26.05±1.68

**Step ** **2**	47.13±2.03	45.78±1.57	45.65±1.78	43.66±3.33

**Step ** **3**	31.66±1.95	32.28±1.78	33.15±1.57	31.15±0.65

**Patients with plantar fasciitis**				

**Step ** **1**	6.62±0.93%**∗∗∗**	7.26±1.01%**∗∗∗**	8.30±1.46%*∗∗∗*	9.45±1.13%*∗∗∗*

**Step ** **2**	57.23±3.51%^**++**^	56.08±3.05%^**++**^	47.50±6.95%	40.34±8.05%

**Step ** **3**	36.14±3.17%	36.64±2.28%	44.10±7.50%^**δ**^	49.19±9.29%^**δ**^

## Data Availability

All data analyzed during this study are included in this publication. The datasets during and/or analyzed during the current study are available from the corresponding author on reasonable request.

## Abbreviations

BF: * Biceps femoris*
BMI: Body Mass Index
EMG: Electromyogram
H-wave: Monosynaptic reflex or Hoffmann's reflex
LFS: Report of the length according to the surface of the statokinesigram
MVC: Maximum Voluntary Contraction
M-wave: Muscle response
RMS: Root Mean Square
SOL: * Soleus*
TA: * Tibialis anterior*
VFY: Variance of the placement of the center of pressure in the sagittal plane
VM: * Vastus medialis*
V-wave: Volitional wave or volley of H-reflex impulses
Xm: Mean placement of the center of pressure in the frontal plane
Ym: Mean placement of the center of pressure in the sagittal plane.
